# The impact of early cranioplasty on neurological function, stress response, and cognitive function in traumatic brain injury

**DOI:** 10.1097/MD.0000000000039727

**Published:** 2024-11-01

**Authors:** Jun Li, Ning Li, Wei Jiang, Aimin Li

**Affiliations:** a Department of Neurosurgery, Lianyungang First People’s Hospital Lianyungang, Jiangsu, China.

**Keywords:** cognitive function, early cranioplasty, efficacy, neurological function, stress response, traumatic brain injury

## Abstract

To analyze the efficacy of early cranioplasty in patients with traumatic brain injury and its impact on neurological function, stress response, and cognitive function. A total of 90 patients with traumatic brain injury admitted to the hospital from January 2021 to March 2024 were included in the study. The patients were divided into an observation group (45 cases) and a control group (45 cases) based on the timing of their cranioplasty. The control group underwent cranioplasty 3 to 6 months post-trauma, while the observation group received cranioplasty within 3 months post-trauma. Neurological function was assessed using the National Institutes of Health Stroke Scale. Cognitive function was evaluated using the Functional Independence Measure, Mini-Mental State Examination, and Neurobehavioral Cognitive Status Examination. Blood samples were collected to measure and compare serum levels of interleukin-6, cortisol, and tumor necrosis factor-alpha between the 2 groups. The observation group demonstrated a higher rate of excellent recovery compared to the control group (95.56% vs 80.00%), with significantly lower National Institutes of Health Stroke Scale scores ([11.18 ± 2.35] vs [14.74 ± 3.61], *P* < .05). Posttreatment scores for Functional Independence Measure, Mini-Mental State Examination, and Neurobehavioral Cognitive Status Examination were significantly higher in the observation group compared to the control group ([59.26 ± 6.12] vs [47.86 ± 5.27], [25.02 ± 4.61] vs [22.74 ± 5.13], [103.52 ± 10.63] vs [88.76 ± 7.39], *P* < .05). Serum levels of interleukin-6, cortisol, and tumor necrosis factor-alpha were significantly lower in the observation group ([22.76 ± 4.15] ng/mL vs [25.38 ± 5.27] ng/mL, [66.29 ± 4.91] nmol/L vs [78.24 ± 6.08] nmol/L, [3.36 ± 1.02] ng/mL vs [4.91 ± 0.98] ng/mL, *P* < .05). The total incidence of postoperative complications was significantly lower in the observation group (8.70% vs 26.09%, *P* < .05). Early cranioplasty is beneficial for the postoperative recovery of patients with traumatic brain injury. It improves neurological function, enhances cognitive function, and reduces stress response, while also significantly lowering the incidence of postoperative complications.

## 1. Introduction

Traumatic brain injury (TBI) is typically caused by direct or indirect external forces to the head, often leading to elevated intracranial pressure and intracranial hematomas, which necessitate large craniectomy for decompression and subsequently result in skull defects. Additionally, skull defects commonly arise from conditions such as cerebrovascular diseases, congenital cerebrovascular malformations, tumor resections, refractory intracranial hypertension, or progressive skeletal disorders following craniectomy, and may also result from congenital skull defects, representing a prominent sequela of craniectomy.^[1,2]^ Patients with skull defects may experience psychological distress due to the visible defect, leading to anxiety, pessimism, depression, and increased psychological burden, which adversely impacts their quality of life. More critically, exposure of brain tissue to atmospheric pressure due to skull defects may result in subcutaneous sinking syndrome, one of the rare complications.^[3–5]^ Therefore, craniectomy for TBI is often accompanied by cranioplasty.

With recent economic development and increased traffic accidents leading to a rise in severe TBI cases, craniectomy and cranioplasty have become routine procedures in neurosurgical practice, significantly benefiting patient outcomes by promoting neurological recovery, and improving cerebral blood flow and cerebrospinal fluid dynamics post-decompression.^[6]^ However, as the procedure evolves, certain drawbacks have emerged. Despite advancements in repair techniques, the incidence of postoperative complications remains relatively high. Common complications include infections, seromas, material loosening, material exposure, and intracranial hematomas.^[7]^ Severe complications not only affect treatment outcomes but also significantly increase the risk of repair failure, thereby exacerbating patient suffering, prolonging recovery, and increasing medical costs.^[7,8]^ Despite these issues, the benefits of cranioplasty following decompressive craniectomy generally outweigh the risks, making it a necessary procedure rather than one to be abandoned. Therefore, minimizing or avoiding postoperative complications, improving treatment outcomes, and accurately predicting patient prognosis have become key focuses for both medical professionals and patients.

It is generally accepted that cranioplasty is performed 3 to 6 months after decompressive craniectomy for TBI.^[6,7]^ During this period, brain tissue damage and intracranial hypertension symptoms typically subside, and the patient’s condition stabilizes. Recent studies have reported that long-term skull defects can lead to imbalances in intracranial space structures, potentially inducing intracranial structural changes and neurological impairments.^[2,8–10]^ Consequently, many neurosurgeons question the traditional delayed cranioplasty timing (3–6 months) and are exploring the advantages of early cranioplasty (1–3 months) regarding complications and neurological outcomes. Therefore, the optimal timing for cranioplasty may be within 1 to 3 months post-injury. This study aims to evaluate the efficacy of early cranioplasty in treating TBI patients and its effects on neurological function, stress responses, and cognitive function. To further investigate the timing of cranioplasty, this study surveyed 90 patients with cranial trauma, with the following results.

## 2. Materials and methods

### 2.1. General information

This retrospective study was approved by the Ethics Committee of Lianyungang First People’s Hospital. A total of 90 patients with TBI admitted to the hospital from January 2021 to March 2024 were selected for the study. The patients were divided into an observation group (45 cases) and a control group (45 cases) based on the timing of treatment. The observation group comprised 26 males and 19 females, aged 27 to 65 years, with a mean age of 42.62 ± 2.64 years. The control group included 24 males and 21 females, aged 29 to 64 years, with a mean age of 33.25 ± 3.06 years.

### 2.2. Inclusion criteria

1 Age ≥18 years.
2 Head trauma with cranial and brain injury confirmed by CT or MRI.
3 Glasgow Coma Scale score >12.
4 The patient had indications for craniotomy decompression.
5 Vital signs are stable; Regular follow-up, complete medical records.
6 The patient or family member gives informed consent and signs informed consent.

### 2.3. Exclusion criteria

1 Complications such as hydrocephalus or other intracranial organic lesions.
2 Presence of psychiatric disorders, abnormal heart.
3 Surgical contraindications.
4 Patients with severe intracranial infection, severe malnutrition and uncontrolled diabetes before operation.
5 Patients with severe liver and kidney dysfunction, malignant tumor and coagulation dysfunction.

There were no statistically significant differences in gender and age between the 2 groups (*P* > .05), indicating comparability.

#### 2.3.1. Sample size

We used the software G*Power to calculate the sample size. Based on previous studies and preexperimental results, a medium effect size Cohen *w* = 0.3 was used as the effect size. In addition, we set the significance level (*α*) at 0.05 and expect the efficacy (1 − *β*) to be 0.80. Taking into account possible missing data and other uncontrollable factors in the experiment, we increased the sample size by 10%. The sample size was finally determined to be 45 people per group. The efficacy analysis showed that the set sample size could reach the expected efficacy level, was sufficient to detect the expected effect, and had sufficient statistical power to support the study’s conclusion.

#### 2.3.2. Clinical symptoms

The primary clinical manifestations in patients included memory decline, intellectual impairment, hearing loss, visual field defects, reduced limb sensation, limb movement disorders, language impairment, epilepsy, and nonspecific symptoms such as headache, dizziness, localized tenderness, irritability, fear, anxiety, sensitivity to noise and vibration, vague discomfort at the defect site, fatigue, depression, inferiority complex, and various other psycho-neurological symptoms associated with cranial injury syndrome. Detailed conditions for both groups are shown in Table 1.

**Table 1:** Analysis of specific clinical manifestations of 2 groups of patients (cases, %).

Clinical manifestation	Observation group (n = 45)	Control group (n = 45)	*X* ^2^	*P*
Memory decline	30 (66.67)	32 (71.11)	0.16	>.005
Intellectual decline	11 (24.44)	13 (28.89)	0.09	>.005
Hearing loss	12 (26.67)	15 (33.33)	0.40	>.005
Visual field defect	8 (17.78)	13 (28.89)	1.16	>.005
Decreased limb sensation	30 (66.67)	35 (77.77)	1.00	>.005
Limb movement disorders	26 (57.77)	30 (66.67)	0.49	>.005
Language dysfunction	20 (44.44)	28 (62.22)	2.18	>.005
Epilepsy	2 (4.44)	1 (2.22)	0.04	>.005
Nonspecific symptoms	43 (95.56)	44 (97.78)	0.04	>.005
Pathological reflex positive	19 (42.22)	21 (46.67)	0.14	>.005

*The *P* values in the table were corrected by Bonferroni, that is, *P* < .005 was considered meaningful.

### 2.4. Methods

The cranioplasty procedure was identical for both groups and involved the following steps: pay close attention to various physiological indications of patients from 3 days before surgery to prepare for surgery. During the operation, patients in both groups were intubated under general anesthesia, disinfected with complex iodine, cut the skin according to the original incision or expanded the original incision, separated the skin flap with disposable electric knife, cut the scalp layer by layer, bluntly separated the temporal muscle and the subcap aponeurotic space, exposed the entire skull defect, cut the periosteal layer along the edge of the bone window and peeled the bone around 2 cm. After separation, the bleeding spots on the medial flap and dural surface were completely hemostatic with double electrodes. The surgical field was repeatedly rinsed with hydrogen peroxide, iodine complex and normal saline. Sterile three-dimensional titanium alloy mesh was selected according to the repair size of the bone window and the three-dimensional CT reconstruction image, and the three-dimensional titanium mesh was fixed to the bone window to avoid periosteal tissue between the titanium mesh and the bone window. The three dimensional titanium mesh was fastened with titanium nails. After the operation, it was sutured layer by layer and wrapped with pressure. Postoperative antibiotics were used to prevent infection, and the drainage tube was removed 4 to 7 days after surgery. The control group underwent cranioplasty 3 to 6 months after operation, and the observation group underwent cranioplasty 3 months after operation. After repair, routine hemostasis and dressing were applied. All surgeries were performed by the same experienced surgical team. If a patient has a damaged dura, the patient’s dura will be repaired and the skull reconstructed.

### 2.5. Observation indicators

1 Clinical efficacy was evaluated using the GCS 1 month after cranioplasty: scores of 13 to 15 were classified as excellent, 9 to <13 as good, and 3 to <9 as poor.
2 Neurological function was assessed using the National Institutes of Health Stroke Scale (NIHSS),^[5]^ with a total score of 42 points; higher scores indicated more severe neurological deficits.
3 Cognitive function was evaluated using the Functional Independence Measure (FIM) scale,^[6]^ the Mini-Mental State Examination (MMSE) scale,^[7]^ and the Neurobehavioral Cognitive Status Examination (NCSE) scale,^[8]^ with higher scores indicating better cognitive function.
4 Blood samples were collected preoperatively and at 3 days postoperatively to measure serum levels of interleukin-6 (IL-6), cortisol (Cor), and tumor necrosis factor-alpha (TNF-α) using enzyme-linked immunosorbent assay.
5 Postoperative complications were compared between the 2 groups.

### 2.6. Statistical analysis

Data were analyzed using SPSS 21.0 statistical software. Normally distributed measurement data were expressed as mean ± standard deviation ($\bar{x}$ ± S) and compared between groups using the *t* test. Categorical data were expressed as counts or percentages and compared using the Chi-square test (*X*²). A difference was considered statistically significant if *P* < .05.

## 3. Results

### 3.1. Comparison of clinical efficacy between the 2 groups

The recovery rate in the observation group was 95.56%, significantly higher than the 80.00% observed in the control group (*P* < .05) (see Table 2).

**Table 2:** Clinical efficacy comparison between the observation and control groups [n (%)].

Group	n	Excellent	Good	Unsatisfactory	Excellent rate
Observation group	45	25 (55.56)	18 (40.00)	2 (4.44)	43 (95.56)
Control group	45	17 (37.78)	19 (42.22)	9 (20.00)	36 (80.00)
*X* ^2^					5.059
*P*					.015

### 3.2. Comparison of NIHSS Scores between the 2 groups

After treatment, the NIHSS scores for the observation group and the control group were 11.18 ± 2.35 and 14.74 ± 3.61, respectively. Both groups exhibited significant reductions from their pretreatment scores (22.07 ± 4.24 for the observation group and 21.49 ± 5.76 for the control group) (*P* < .05). Additionally, the posttreatment NIHSS score in the observation group was significantly lower than that in the control group (*P* < .05) (see Table 3 for detailed results).

**Table 3:** Comparison of NIHSS scores between 2 groups of patients ($\bar{x}$ ± s, points).

Group	n	Before treatment	After treatment
Observation group	45	22.07 ± 4.24	11.18 ± 2.35*
Control group	45	21.49 ± 5.76	14.74 ± 3.61*
*t*		0.470	5.605
*P*		.721	<.001

Compared with before treatment in the same group.

NIHSS = National Institutes of Health Stroke Scale.

* *P* < .05; *P* < .05.

### 3.3. Comparison of FIM, MMSE, and NCSE scores between the 2 groups

After treatment, the FIM, MMSE, and NCSE scores for the observation group were 59.26 ± 6.12, 25.02 ± 4.61, and 103.52 ± 10.63, respectively. In the control group, the posttreatment scores were 47.86 ± 5.27, 22.74 ± 5.13, and 88.76 ± 7.39, respectively. Both groups showed significant improvements in FIM, MMSE, and NCSE scores compared to their pretreatment values. Additionally, scores in the observation group were significantly higher than those in the control group (*P* < .05) (see Table 4 for detailed results).

**Table 4:** Comparison of scores on the FIM, MMSE, and NCSE scales between 2 groups of patients ($\bar{x}$ ± s, points).

Group	n	FIM scale		MMSE scale		NCSE scale	
		Before treatment	After treatment	Before treatment	After treatment	Before treatment	After treatment
Observation group	45	35.26 ± 4.94	59.26 ± 6.12*	18.13 ± 3.94	25.02 ± 4.61*	52.06 ± 3.85	103.52 ± 10.63*
Control group	45	36.15 ± 4.56	47.86 ± 5.27*	18.69 ± 4.17	22.74 ± 5.13*	51.97 ± 4.23	88.76 ± 7.39*
*t*		0.898	9.573	0.662	2.242	0.107	7.732
*P*		.372	<.001	.561	.027	.895	<.001

Compared to before treatment.

FIM = Functional Independence Measure, MMSE = Mini-Mental State Examination, NCSE = Neurobehavioral Cognitive Status Examination.

* *P* < .05.

### 3.4. Comparison of IL-6, Cor, and TNF-α Levels between the 2 groups

After treatment, the serum levels of IL-6, cortisol (Cor), and TNF-α in the observation group were 22.76 ± 4.15 ng/mL, 66.29 ± 4.91 nmol/L, and 3.36 ± 1.02 ng/mL, respectively. In the control group, the posttreatment levels were 25.38 ± 5.27 ng/mL, 78.24 ± 6.08 nmol/L, and 4.91 ± 0.98 ng/mL, respectively. Both groups showed higher levels of IL-6, Cor, and TNF-α after treatment compared to before, but the levels in the observation group were significantly lower than those in the control group (*P* < .05) (see Table 5).

**Table 5:** Comparison of IL-6, Cor, and TNF-α levels between 2 groups of patients ($\bar{x}$ ± s).

Group	n	IL-6 (ng/mL)		Cor (nmol/L)		TNF-α (ng/mL)	
		Before treatment	After treatment	Before treatment	After treatment	Before treatment	After treatment
Observation group	45	18.63 ± 3.49	22.76 ± 4.15*	61.23 ± 3.79	66.29 ± 4.91*	2.45 ± 0.63	3.36 ± 1.02*
Control group	45	17.59 ± 4.06	25.38 ± 5.27*	60.94 ± 4.12	78.24 ± 6.08*	2.51 ± 0.46	4.91 ± 0.98*
*t*		1.317	2.649	0.351	10.371	0.522	7.432
*P*		.153	.010	.726	<.001	.620	<.001

Compared to before treatment.

Cor = cortisol, IL-6 = interleukin-6, TNF-α = tumor necrosis factor-alpha.

* *P* < .05.

### 3.5. Comparison of postoperative complications between the 2 groups

The total incidence of postoperative complications in the observation group was 8.89%, significantly lower than the 26.67% observed in the control group (*P* < .05) (see Table 6).

**Table 6:** Comparison of postoperative complications between 2 groups of patients [n (%)].

Group	n	Intracranial infection	Intracranial hemorrhage	Subcutaneous necrosis	Subcutaneous effusion	Chewing discomfort	Incidence rate
Observation group	45	2 (4.44)	0 (0.00)	1 (2.22)	0 (0.00)	1 (2.22)	4 (8.89)
Control group	45	4 (8.89)	1 (2.22)	2 (4.44)	3 (6.67)	2 (4.44)	12 (26.67)
X*							4.842
*P*							.028

## 4. Discussion

TBI is associated with high rates of disability, mortality, and poor prognosis, posing a significant challenge in neurosurgery. Many TBI patients experience complications such as hydrocephalus and brain swelling, which lead to elevated intracranial pressure and further neurological impairment. Delayed decompression can not only increase the risk of mortality but also result in a poor prognosis. Consequently, clinical management guidelines prioritize ventriculoperitoneal shunting for patients with severe TBI and significant intracranial hypertension. This procedure reduces cerebrospinal fluid volume, alleviates brain tissue swelling, and consequently lowers intracranial pressure, which significantly improves patient outcomes.

However, there is ongoing debate about whether early cranial bone repair should be performed immediately following ventriculoperitoneal shunting. Traditionally, it has been argued that in severe TBI cases, the patient’s unstable condition may lead to recurrent intracranial pressure elevation. Keeping the cranial bone site open allows for easier access for future salvage surgeries, potentially saving time and improving rescue success rates. Conversely, leaving the cranial site open can increase psychological stress due to the lack of cranial closure and may decrease brain tissue stability due to external factors such as atmospheric pressure, gravitational forces, and traction on the skin flap, which could impair neurological function.

Optimal recovery time for TBI patients is generally considered to be within 2 months post-injury. Delaying cranial bone repair beyond this period can increase the risk of neurological deficits, leading to a noticeable decline in behavioral capacity and overall quality of life.^[11–13]^ This condition imposes significant psychological and economic burdens on patients, who may also face risks of injury or complications due to inadequate cranial protection. Therefore, timely and effective treatment is crucial.

Current standard treatments for TBI often include decompressive craniectomy and hematoma evacuation, followed by cranial reconstruction after hematoma clearance. This approach facilitates postoperative recovery and enhances clinical outcomes and psychological well-being.^[14]^ However, the optimal timing for cranial reconstruction remains controversial. Some studies suggest that the ideal timing for this procedure is between 3 and 6 months post-injury.^[15]^ Recent reports indicate that prolonged post-traumatic cranial defects can affect cerebral vascular compensation and brain tissue metabolism, potentially leading to oxidative stress, cellular apoptosis, and cytotoxic edema, which can result in neuronal damage and neurological deficits.^[16–20]^

The mechanism of cranioplasty to improve nerve function is not completely clear, and some studies speculate that it is related to abnormal perfusion and cerebral hemodynamic improvement after cranioplasty. Previous studies have established a link between the duration of cranial defects and neurological outcomes in TBI patients, with longer durations being associated with poorer neurological recovery.^[21]^ Our study supports these findings, This suggests that early intervention is advantageous for postoperative recovery and is clinically safe. Specifically, our results indicate that the observation group had significantly lower NIHSS scores posttreatment compared to the control group (*P* < .05), In the past, it was considered that the operation time of secondary cranioplasty after DC was 3 to 6 months. However, Skull repair within 3 months after Decompressive Craniectomy has a good effect. The analysis is that skull repair surgery can restore the integrity and tightness of the skull, and the intracranial environment of patients tends to be normal, which can relieve the pressure on the brain tissue caused by the compression of the skull bone window, improve the blood supply of brain tissue, and promote the return of brain tissue to normal. The dural interface peeling wound is smaller, which can reduce the damage to the nerve function of patients, and make the recovery of the nerve function of patients faster, so that patients can obtain a good prognosis.

The effect of cranioplasty on cognitive and neurological function has been widely recognized by scholars, but the effect of cranioplasty timing on cognitive function remains controversial. Some scholars believe that the neurological prognosis after cranioplasty is the result of a variety of confounding factors. Moreover, the observation group had significantly higher scores on the FIM, MMSE, and NCSE scales posttreatment compared to the control group (*P* < .05). This indicates that early cranial reconstruction surgery enhances cognitive function, likely by facilitating memory and orientation recovery and reducing complications such as cerebral edema and infections.^[23]^ Additionally, early cranial reconstruction has been reported to significantly improve patient prognosis and activities of daily living.^[11,24]^ This aligns with research indicating that early cranial reconstruction contributes to improved prognosis, reduced stress responses, and enhanced neurological recovery.^[25]^ Furthermore, it has been noted that early cranial reconstruction improves cognitive function and stress response in elderly TBI patients, thereby decreasing postoperative complications. As a retrospective study, this study still has some limitations. First, there is selection bias in clinical data, which is inevitable, and patients are heterogeneous, which does not affect the results of this paper. Second, although we have calculated the sample size, it only satisfies statistical significance, and for retrospective studies, the sample size should still be expanded. In future studies, we plan to conduct a randomized controlled trial with multi-medical center cooperation, and increase the relationship between other indicators such as thyroxine, tumor necrosis factor, IL-6 and stress response and cognitive function in patients after early cranioplasty.^[26]^

## 5. Conclusion

In summary, early cranial reconstruction surgery significantly facilitates postoperative recovery, enhances neurological and cognitive function, and reduces stress response in patients with TBI.

## Author contributions

**Conceptualization:** Jun Li, Ning Li, Aimin Li.

**Data curation:** Jun Li, Ning Li, Wei Jiang, Aimin Li.

**Formal analysis:** Jun Li, Ning Li, Wei Jiang, Aimin Li.

**Investigation:** Jun Li, Aimin Li.

**Methodology:** Jun Li, Ning Li, Wei Jiang.

**Writing—original draft:** Jun Li, Aimin Li.

**Writing—review & editing:** Jun Li, Aimin Li.

**Supervision:** Ning Li, Wei Jiang.

**Validation:** Ning Li, Wei Jiang.

**Funding acquisition:** Aimin Li.

## References

[1] Piedra MP Ragel BT Dogan A Coppa ND Delashaw JB. Timing of cranioplasty after decompressive craniectomy for ischemic or hemorrhagic stroke. J Neurosurg. 2013;118:109–14. 23140156 10.3171/2012.10.JNS121037

[2] Ozoner B. Cranioplasty following severe traumatic brain injury: role in neurorecovery. Curr Neurol Neurosci Rep. 2021;21:62. 34674047 10.1007/s11910-021-01147-6

[3] Englar KM Kordahi AM Brandel MG. Application of antibiotic-impregnated polymethyl-methacrylate bone cement for the treatment of infected cranioplasties: initial experience. Ann Plast Surg. 2022;88(4 Suppl 4):S357–60. 37740468 10.1097/SAP.0000000000003079

[4] Shay T Mitchell K-A Belzberg M. Translucent customized cranial implants made of clear polymethylmethacrylate: an early outcome analysis of 55 consecutive cranioplasty cases. Ann Plast Surg. 2020;85:e27–36. 33170582 10.1097/SAP.0000000000002441

[5] Thimukonda Jegadeesan J Baldia M Basu B. Next-generation personalized cranioplasty treatment. Acta Biomater. 2022;154:63–82. 36272686 10.1016/j.actbio.2022.10.030

[6] Champeaux C Froelich S Caudron Y. Titanium three-dimensional printed cranioplasty for fronto-nasal bone defect. J Craniofac Surg. 2019;30:1802–5. 31022139 10.1097/SCS.0000000000005493

[7] Piazza M Grady MS. Cranioplasty. Neurosurg Clin N Am. 2017;28:257–65. 28325460 10.1016/j.nec.2016.11.008

[8] Ye J Zhang W Ye M. Using titanium mesh to replace the bone flap during decompressive craniectomy: a medical hypothesis. Med Hypotheses. 2019;129:109257. 31371088 10.1016/j.mehy.2019.109257

[9] Alkhaibary A Alharbi A Alnefaie N Oqalaa Almubarak A Aloraidi A Khairy S. Cranioplasty: a comprehensive review of the history, materials, surgical aspects, and complications. World Neurosurg. 2020;139:445–52. 32387405 10.1016/j.wneu.2020.04.211

[10] Frassanito P Fraschetti F Bianchi F Giovannenze F Caldarelli M Scoppettuolo G. Management and prevention of cranioplasty infections. Childs Nerv Syst. 2019;35:1499–506. 31222447 10.1007/s00381-019-04251-8

[11] Eaton JC Greil ME Nistal D. Complications associated with early cranioplasty for patients with traumatic brain injury: a 25-year single-center analysis. J Neurosurg. 2022;137:776–81. 35061995 10.3171/2021.11.JNS211557

[12] Tsianaka E Singh A Drosos E Fountas K. Direct consequences of cranioplasty to the brain: intracranial pressure study. J Craniofac Surg. 2021;32:2779–83. 34727479 10.1097/SCS.0000000000007945

[13] Shepetovsky D Mezzini G Magrassi L. Complications of cranioplasty in relationship to traumatic brain injury: a systematic review and meta-analysis. Neurosurg Rev. 2021;44:3125–42. 33686551 10.1007/s10143-021-01511-7 PMC8592959

[14] Lin CH Yang J-T Wang T-C Lin MH Cheng W-C Lee M-H. Is preoperative brain midline shift a determinant factor for neurological improvement after cranioplasty? J Formos Med Assoc. 2015;114:577–82. 24113352 10.1016/j.jfma.2013.09.008

[15] Su JH Wu Y-H Guo N-W. The effect of cranioplasty in cognitive and functional improvement: experience of post traumatic brain injury inpatient rehabilitation. Kaohsiung J Med Sci. 2017;33:344–50. 28738975 10.1016/j.kjms.2017.05.002 PMC11916026

[16] Weis CN Webb EK deRoon-Cassini TA Larson CL. Emotion dysregulation following trauma: shared neurocircuitry of traumatic brain injury and trauma-related psychiatric disorders. Biol Psychiatry. 2022;91:470–7. 34561028 10.1016/j.biopsych.2021.07.023 PMC8801541

[17] Cash A Theus MH. Mechanisms of blood-brain barrier dysfunction in traumatic brain injury. Int J Mol Sci. 2020;21:3344. 32397302 10.3390/ijms21093344 PMC7246537

[18] Zhang S Wu X Wang J. Adiponectin/AdiopR1 signaling prevents mitochondrial dysfunction and oxidative injury after traumatic brain injury in a SIRT3 dependent manner. Redox Biol. 2022;54:102390. 35793583 10.1016/j.redox.2022.102390 PMC9287731

[19] Wang H Zhou X-M Wu L-Y. Aucubin alleviates oxidative stress and inflammation via Nrf2-mediated signaling activity in experimental traumatic brain injury. J Neuroinflammation. 2020;17:188. 32539839 10.1186/s12974-020-01863-9 PMC7294631

[20] Lin Y Zhang J Lu D. Uqcr11 alleviates oxidative stress and apoptosis after traumatic brain injury. Exp Neurol. 2023;370:114582. 37884186 10.1016/j.expneurol.2023.114582

[21] Yang J Sun T Yuan Y Li X Yu H Guan J. Evaluation of titanium cranioplasty and polyetheretherketone cranioplasty after decompressive craniectomy for traumatic brain injury: a prospective, multicenter, non-randomized controlled trial. Medicine (Baltim). 2020;99:e21251. 10.1097/MD.0000000000021251 PMC7386959 32791701

[22] Corallo F De Cola MC Lo Buono V. Early vs late cranioplasty: what is better? Int J Neurosci. 2017;127:688–93. 27609482 10.1080/00207454.2016.1235045

[23] Kim BW Kim TU Hyun JK. Effects of early cranioplasty on the restoration of cognitive and functional impairments. Ann Rehabil Med. 2017;41:354–61. 28758072 10.5535/arm.2017.41.3.354 PMC5532340

[24] Zheng F Xu H von Spreckelsen N. Early or late cranioplasty following decompressive craniotomy for traumatic brain injury: a systematic review and meta-analysis. J Int Med Res. 2018;46:2503–12. 29779445 10.1177/0300060518755148 PMC6124291

[25] Xu H Niu C Fu X. Early cranioplasty vs. late cranioplasty for the treatment of cranial defect: a systematic review. Clin Neurol Neurosurg. 2015;136:33–40. 26056810 10.1016/j.clineuro.2015.05.031

[26] Di Stefano C Rinaldesi ML Quinquinio C. Neuropsychological changes and cranioplasty: a group analysis. Brain Inj. 2016;30:164–71. 26647093 10.3109/02699052.2015.1090013

